# Primary Colonic Leiomyosarcoma Initially Diagnosed as Kaposi Sarcoma: A Case Report

**DOI:** 10.7759/cureus.112974

**Published:** 2026-07-19

**Authors:** Ana Y Sandoval-Mussi, Dioselina Lanzagorta-Ortega, Ángela M Pérez-Paredes, Paris A Cisneros-Ramos, Saulo Mendoza-Ramírez, Enrique R Jean-Silver, Roberto R Hernández-Peña, Jorge A Ortiz de la Peña-Rodríguez

**Affiliations:** 1 General Surgery, American British Cowdray Medical Center, Mexico City, MEX; 2 Medicine and Surgery, Tecnológico de Monterrey, Mexico City, MEX; 3 Pathology, American British Cowdray Medical Center, Mexico City, MEX; 4 Clinical Sciences, Tecnológico de Monterrey, Escuela de Medicina y Ciencias de la Salud, Mexico City, MEX; 5 Oncology Surgery, American British Cowdray Medical Center, Mexico City, MEX

**Keywords:** case report, colon cancer, colonic kaposi sarcoma, gastrointestinal stromal tumor (gist), immunohistochemistry, smooth muscle tumor

## Abstract

Primary colonic leiomyosarcomas are relatively uncommon mesenchymal tumors, often posing diagnostic challenges due to their overlapping features with other gastrointestinal tumors. In this article, we report the case of a 61-year-old male who presented with abdominal pain and weight loss, whose initial endoscopic biopsy was interpreted as colonic Kaposi sarcoma, with tumor-cell positivity for cluster of differentiation 34 and human herpesvirus 8. Further surgical intervention, characterized by a right radical hemicolectomy and histopathological analysis, revealed a primary colonic leiomyosarcoma. This case highlights the diagnostic complexity associated with colonic leiomyosarcomas, the importance of comprehensive histopathological assessment, and the need for timely surgical management. The clinical course of the patient analyzed illustrates how misclassification can alter therapeutic strategies, underscoring the value of a timely and accurate diagnosis in guiding appropriate oncological management.

## Introduction

Leiomyosarcomas are rare, aggressive tumors that originate from smooth muscle cells, located in the gastrointestinal tract in only 20% of cases. They account for only 2% of all gastrointestinal cancers and approximately 0.1% of colorectal malignancies. Gastrointestinal leiomyosarcomas present more commonly in men, in their fifth to sixth decade of life, and they are most frequently found in the stomach and small intestine, followed by the colon and the rectum [1,2]. Regarding their histological characteristic features, leiomyosarcomas are typically high-grade tumors made up of spindle-shaped cells that show frequent mitotic activity and resemble smooth muscle cells, featuring elongated, cigar-shaped nuclei and eosinophilic cytoplasm [3]. Their clinical and pathological features often overlap with those of gastrointestinal stromal tumors (GISTs), historically leading to their misclassification, particularly before the use of immunohistochemistry [4,5].

The diagnosis, staging, and management of colonic leiomyosarcomas are challenging due to a wide variety of differential diagnoses and a lack of data describing the true demographic and clinical features of the disease. Due to their rarity, colonic leiomyosarcomas are most often reported as isolated case reports, which limits the possibility of establishing standardized diagnostic or therapeutic guidelines. As a result, management strategies are generally based on individual cases rather than evidence-based protocols [6]. According to reported cases, radical surgery remains the treatment of choice, as high recurrence rates have been observed in patients who undergo only local excision. In cases of metastatic disease or high-grade tumors, surgery is often preceded by an anthracycline-based chemotherapy regimen. However, the prognosis is highly individualized due to the limited amount of available data surrounding this rare pathology [7]. Therefore, in large colonic spindle-cell tumors, limited biopsy material may lead to diagnostic misclassification, and definitive diagnosis may require broad immunohistochemical evaluation of the resected specimen.

In this article, we present the case of a 61-year-old male with a history of colonic Kaposi sarcoma who underwent right hemicolectomy, with histopathological analysis revealing a colonic leiomyosarcoma. We highlight the diagnostic challenges of this rare entity through the correlation of imaging, endoscopic, intraoperative, and pathological findings, underscoring its implications for clinical management.

## Case presentation

A 61-year-old man, with no relevant medical history, began experiencing diffuse pain in the left hemiabdomen in October 2023. He consulted a gastroenterologist and underwent routine tests, which revealed a positive occult blood test result. Later, a colonoscopy was performed in November 2023, reporting ulcerative colitis (UC) with chronic nonspecific proctitis. The original endoscopic images and pathology material were unavailable for review; therefore, UC could not be independently confirmed and was not considered an established diagnosis at our institution. The patient received hydroxychloroquine and pargeverine outside our hospital, with partial symptomatic improvement. The indication for hydroxychloroquine was not documented, and no confirmed autoimmune disease was identified in the records available to us.

**Figure 1:**
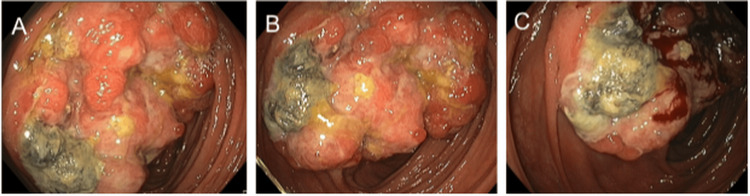
Second colonoscopy. Neoplastic-appearing mass in the cecum and ascending colon, occluding approximately 80% of the lumen, with friable mucosa, nodular surface, ischemic areas (A and B), and (C) active bleeding.

In March 2024, the patient presented to our institution because of worsening symptoms and an unintentional weight loss of approximately 10 kg. A second colonoscopy demonstrated a large cecal and ascending-colon mass (Figure 1). Limited biopsy material was interpreted as Kaposi sarcoma without cutaneous involvement. The tumor cells were positive for cluster of differentiation 34 (CD34) and human herpesvirus 8 (HHV-8), with a Ki-67 proliferation index of approximately 80%.

Laboratory tests indicated immunosuppression characterized by lymphopenia, low absolute CD3 (662) and CD4 (372) levels, with low total immunoglobulin G (585) and its subclasses two (98), three (10.7), and four (<1); however, human immunodeficiency virus (HIV) antigens and viral load were negative, and tumor markers were negative.

**Figure 2:**
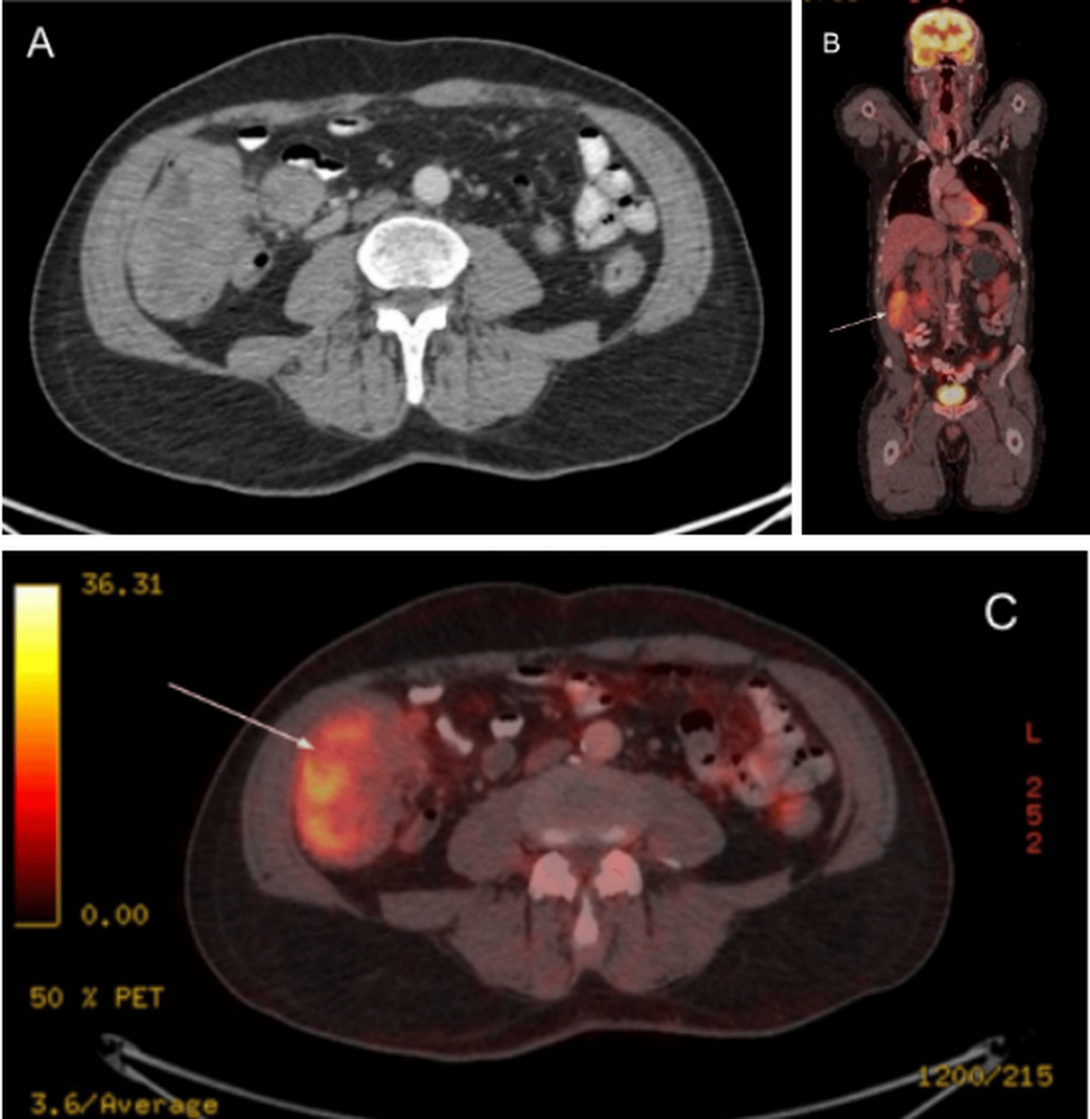
Positron emission tomography-computed tomography scan. (A) Normal small bowel loops. (B and C) Cecal solid heterogeneous lesion measuring 83 mm (maximum standardized uptake value: 7.1) with necrosis and photopenia (white arrows); associated perilesional lymphadenopathies up to 36 × 40 mm (maximum standardized uptake value: 4.7).

A positron emission tomography-computed tomography (PET-CT) scan revealed a metabolic lesion located in the cecum compatible with neoplastic activity and multiple perilesional metabolic adenopathies consistent with secondary neoplastic deposits (Figure 2).

**Figure 3:**
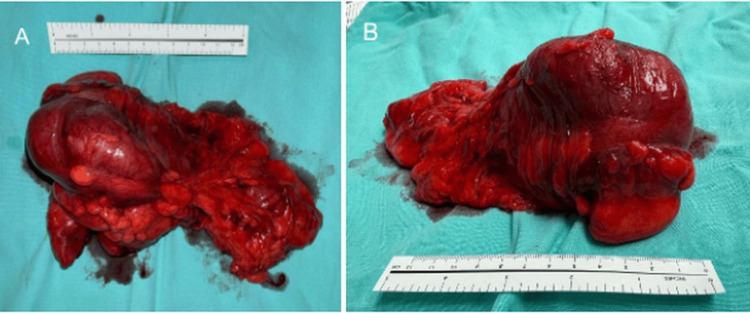
Right radical hemicolectomy. (A and B) Right hemicolectomy specimen showing a firm, thickened mass involving the cecum and ascending colon, measuring approximately 15 cm.

Consequently, the patient underwent a right radical hemicolectomy (Figure 3) with side-to-side ileocolic anastomosis.

**Figure 4:**
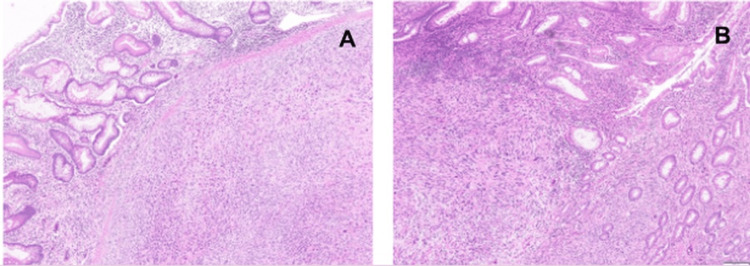
Intestinal leiomyosarcoma. (A) Low-power hematoxylin and eosin-stained section showing a submucosal colonic neoplasm (original magnification ×40). (B) Higher-power view demonstrating intersecting fascicles of spindle cells (original magnification ×100).

The histopathological report revealed an 8.5 cm × 7.0 cm high-grade pleomorphic leiomyosarcoma occluding 90% of the bowel lumen, located in the ileocecal valve (Figure 4).

**Figure 5:**
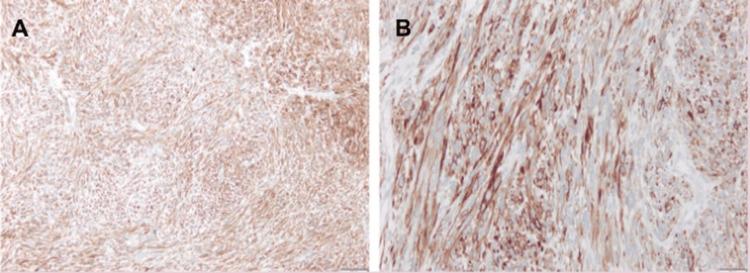
Intestinal leiomyosarcoma immunostains. (A) Diffuse caldesmon expression in the resected primary tumor (original magnification ×100). (B) Higher-power view showing cytoplasmic caldesmon positivity in neoplastic spindle cells (original magnification ×200).

Immunostains (HHV8, ETS-related gene (ERG), CD34, discovered on GIST-1 (DOG-1), cytomegalovirus (CMV), Epstein-Barr virus-encoded RNA (EBER), John Cunningham (JC) virus) were negative with Ki67 (80%) and caldesmon reactive (Figure 5, Table 1). Out of 27 lymph nodes, 10 were positive for metastatic leiomyosarcoma, 60 mitoses were identified in 5 mm², and necrosis was present in 10% of the tumor.

**Table 1:** Comparison of immunohistochemical findings in the initial biopsy and surgical resection specimen. HHV-8 = human herpesvirus 8; CD34 = cluster of differentiation 34; Ki-67 = Ki-67 proliferation index; ERG = ETS-related gene; DOG-1 = discovered on GIST-1; CMV = cytomegalovirus; EBER = Epstein-Barr virus-encoded RNA; JC virus = John Cunningham virus

Marker	Initial biopsy (Kaposi sarcoma)	Surgical specimen (leiomyosarcoma)
HHV-8	Positive	Negative
CD34	Positive	Negative
Caldesmon	Not performed	Positive
Ki-67	80%	80%
ERG	Not performed	Negative
DOG-1	Not performed	Negative
CMV	Not performed	Negative
EBER	Not performed	Negative
JC Virus	Not performed	Negative

The patient had an uneventful postoperative course and was discharged home six days after the surgery, receiving adjuvant chemotherapy with doxorubicin 20 mg/m² for three administrations.

## Discussion

Leiomyosarcomas are rare malignant mesenchymal tumors of smooth muscle origin, most likely to occur in middle-aged males. About 20% occur in the gastrointestinal tract, representing only 2% of gastrointestinal malignancies. Primary colonic leiomyosarcomas are less common, accounting for 0.1% of colon malignancies (1 in 100,000 patients) [1,2]. They usually infiltrate the mucosa, causing ulceration and necrosis, and histologically are high-grade tumors composed of spindle cells with nuclear atypia and high mitotic activity. Staining is positive for smooth muscle markers (actin, desmin, vimentin, h-caldesmon) and negative for CD117, CD34, KIT, and DOG1, some of which were observed in the presented case [3-5].

The typical initial symptoms of presentation are nonspecific, presenting as incidental diagnosis, commonly with abdominal pain and gastrointestinal bleeding, and not so commonly with weight loss, tenesmus, changes in bowel habits, and vomiting [2,5]. Gastrointestinal leiomyosarcomas are considered aggressive as they tend to metastasize to the lung, peritoneum, liver, and, rarely, to lymph nodes. Minimal criteria for malignancy have not been established; however, malignant features include a minimum mitotic rate of 1 to 4 per 10 high-power fields, nuclear atypia, and the presence of coagulative necrosis [5].

Endoscopically, they may present as intraluminal polypoid masses located in the small intestine or colon, being frequently misdiagnosed as GISTs. Regarding the misdiagnosis of leiomyosarcomas, it is important to note that GISTs were previously wrongfully classified as leiomyosarcomas [4]. Biomarkers are highly useful in differentiating GISTs from other types of smooth muscle neoplasms, as leiomyosarcomas are positive for muscle markers and GISTs for KIT, CD34, CD117, and DOG1. Desmin shows higher specificity because GISTs may exhibit α-smooth muscle actin staining [1].

Smooth muscle neoplasms also occur in association with immunodeficiency states, leading to a wide variety of differential diagnoses. Leiomyosarcomas may be confused with the spindle cell-predominant type of Kaposi sarcoma [8]. Regarding the relationship between this type of malignancy and immunosuppressed patients, there have been reported cases of leiomyosarcomas in patients with HIV and those immunosuppressed after receiving organ transplants. A possible association between the immunosuppressive therapy given to patients with UC and this type of malignancy has not been shown, and therefore, the type of therapy and therapeutic window have not been studied. Nevertheless, impaired immune surveillance, the response to foreign allograft antigens, and the vulnerability of immunosuppressed patients to viral infections with malignant potential may lead to delays in diagnosis and mismanagement, resulting in poor clinical outcomes [3]. This is relevant, as the initial biopsy showed positivity for CD34 and HHV-8, whereas the resected specimen demonstrated smooth muscle differentiation with caldesmon positivity and negative HHV-8/ERG/CD34/DOG-1, supporting the diagnosis of a leiomyosarcoma.

The cornerstone treatment for localized colonic leiomyosarcoma is surgical resection with negative margins (R0), ideally performed within a multidisciplinary setting. Depending on the tumor location, hemicolectomy is often indicated, with en bloc resection of adjacent structures with tumor-free margins [9,10]. For patients with metastatic disease, first-line chemotherapy is based on anthracyclines, particularly doxorubicin, either as a single agent or in combination with ifosfamide, trabectedin, or dacarbazine. Complete remission is infrequent regardless of the agent used, presenting resistance to chemotherapy and radiotherapy [11]. Nodal sampling or dissection is reserved for cases with radiological or intraoperative suspicion of nodal disease. Achieving R0 resection is the most relevant prognostic factor; precise tumor staging follows the American Joint Committee on Cancer system for soft-tissue sarcomas, which includes tumor size, grade, and metastatic status. Furthermore, validated nomograms can be utilized to personalize risk assessment and guide clinical decision-making, particularly in the context of adjuvant therapies. These parameters guide prognosis, adjuvant therapy considerations, and follow-up strategies [12,13].

Population-based data suggest that colorectal leiomyosarcoma has an approximately 51.6% five-year overall survival rate, although estimates are limited by the rarity and heterogeneity of the disease. Follow-up is key, using PET-CT scan every four to six months in the first three to five years for low-grade cases with a distant metastasis rate of 10%, compared to follow-up every three months for high-grade cases with a distant metastasis rate of 50% [14] and a local recurrence rate of 50%. Poor prognostic factors are older adults, tumoral necrosis, high tumor grade (III/IV), distant metastasis, and tumor size ≥5 cm [15].

## Conclusions

This case highlights the diagnostic complexities and therapeutic challenges associated with colonic leiomyosarcomas, a rare malignancy often misclassified due to its histopathological similarities with GISTs. Limited endoscopic biopsy material led to an initial diagnosis of Kaposi sarcoma, whereas examination of the resected specimen demonstrated smooth muscle differentiation and a discordant immunophenotype that established leiomyosarcoma. Accurate diagnosis of colonic leiomyosarcomas is essential for appropriate management, as demonstrated by the need for radical surgical intervention and adjuvant chemotherapy in this case. Furthermore, the association between immunosuppression and smooth muscle neoplasms, as observed in this patient, suggests a need for vigilant long-term monitoring in immunocompromised individuals to ensure timely identification and treatment of such malignancies. The immune abnormalities observed in this patient may have contributed to diagnostic complexity, but a causal relationship with tumor development cannot be inferred from a single case. This case contributes to the limited literature on colonic leiomyosarcomas and emphasizes the importance of distinguishing these tumors from other gastrointestinal neoplasms to optimize patient outcomes.
